# Blood cortisol and faecal cortisol metabolite concentrations following an ACTH challenge in unanaesthetized brown bears (*Ursus arctos*)

**DOI:** 10.1093/conphys/coae093

**Published:** 2025-01-15

**Authors:** Justin A Piñero, Heiko T Jansen, Charles T Robbins, Ellery P Vincent, Diana J R Lafferty

**Affiliations:** Wildlife Ecology and Conservation Science Lab, Department of Biology, Northern Michigan University, 1401 Presque Isle Ave, Marquette, MI 49855-5301, USA; Department of Integrative Physiology and Neuroscience, College of Veterinary Medicine, Washington State University, 255 E Main Street, Pullman, WA 99164-7010, USA; School of the Environment and School of Biological Sciences, Washington State University, 255 E Main Street Pullman, WA 99164-2812, USA; School of the Environment and School of Biological Sciences, Washington State University, 255 E Main Street Pullman, WA 99164-2812, USA; Wildlife Ecology and Conservation Science Lab, Department of Biology, Northern Michigan University, 1401 Presque Isle Ave, Marquette, MI 49855-5301, USA

**Keywords:** Elisa assay, endocrinology, enzyme-linked immunosorbent assay, glucocorticoids, hormone, hormone challenge, stress

## Abstract

Faecal cortisol metabolites (FCMs) are increasingly used to index physiological stress in wildlife. Cortisol and other stress hormones act to mobilize glucose, providing energy for the organism to respond to environmental perturbations. Cortisol, the predominant glucocorticoid (GC) in most mammals, is metabolized by the liver and excreted as FCMs. For FCMs to serve as a meaningful physiological index of stress in brown bears (*Ursus arctos*), we sought to quantify the relationship between blood cortisol and FCM concentrations. Consequently, we conducted an adrenocorticotropic hormone (ACTH) challenge on nine unanaesthetized captive brown bears at the Washington State University Bear Research, Education, and Conservation Center. We collected 10 ml of blood at 0, 3, 6, 24, 48 and 72 h post-injection to measure changes in blood cortisol concentrations. Faecal samples were collected between 7:00 am and 8:00 pm from 24 h prior to injection through 72 h post ACTH challenge. We found that FCM concentration was positively correlated with blood cortisol concentrations and that peak blood cortisol concentrations occurred between 3 and 6 h following an ACTH challenge, whereas FCMs peaked between 10 and 27 h after injection.

## Introduction

Wildlife depends on a variety of internal and external cues to adaptatively respond to changing conditions. In vertebrates, environmental cues activate the hypothalamic–pituitary–adrenal (HPA) axis, which stimulates the release of cortisol and other glucocorticoids (GCs) from the adrenal cortex to help individuals meet the demands imposed by environmental stressors (Mondol *et al.,* 2020). For instance, the autonomic nervous system provides immediate energy to respond to acute environmental pressure (e.g. ‘fight or flight response’), whereas the HPA axis provides a longer term response to environmental pressures (Adamo, 2014; Romero and Gormally, 2019). Whilst short-term HPA axis activation facilitates adaptive responses to environmental stress, chronic HPA axis activation can have detrimental health effects including immune suppression, muscle wasting, weight loss and the reduction or loss of reproduction (Charbonnel *et al.,* 2008).

In most mammals, cortisol is the predominant GC secreted in the blood in response to a stressor (Romero, 2004). As such, elevated blood cortisol concentrations can provide a quantitative means for evaluating physiological condition in animals (von der Ohe and Servheen, 2002). Cortisol and cortisol metabolites are often measured using enzyme-linked immunoassays (ELISAs) in which specific antibodies bind with GCs and GC metabolites, allowing for quantification (Möstl *et al.,* 2005). Cortisol can be extracted from a variety of animal matrices including plasma, which requires more invasive procedures, whereas hair, feathers, saliva and faeces allow for non-invasive opportunities to obtain samples for the purpose of analysing or indexing stress hormones (Palme, 2012). Further, blood cortisol concentrations provide insight into the physiological state of an organism at a single point in time, which can be highly variable based on time of day, diet or a recent stressful event (Touma *et al.,* 2003; Davies *et al.,* 2013). Additionally, use of blood measures of cortisol can be restrictive as animals must be captured first and occasionally chemically immobilized, potentially inducing an organism’s stress response to capture and restraint stress (Millspaugh and Washburn, 2004; Thompson *et al.,* 2020).

Whilst cortisol can be measured directly in blood, circulating cortisol is metabolized by the liver by A-ring reductases and 11beta hydroxysteroid dehydrogenases resulting in little cortisol or other native GCs to be found in faeces (Finken *et al.,* 1999). The resulting cortisol metabolites are excreted into the intestine via bile, and later excreted in both urine and faeces (Di Francesco *et al.,* 2021). Therefore, there is a time delay between peak blood cortisol concentrations following release from the adrenal gland and faecal metabolite concentrations dependent on the rate of hepatic cortisol metabolism, length of the intestinal tract, secretion of GC metabolites into the intestinal tract, presence of food and rate of food residue passage (Touma and Palme, 2005). Importantly, rather than a single moment in time, faecal cortisol metabolites (FCMs) provide an integrated measure of fluctuating blood cortisol concentrations from the time FCMs are formed to when FCMs are excreted. Faecal samples can also be obtained non-invasively, thereby removing potential bias occurring as a result of animal capture and handling stress (Möstl and Palme, 2002). Whilst FCMs may decline significantly within some faecal samples within the first 24 h following deposit, multiple studies have observed significant changes in FCM after 24 h of exposure (Stetz *et al.,* 2013; Dalerum *et al.,* 2020). FCMs can be influenced by diet, season, food availability, sex, sample exposure time and reproductive status (Babic *et al.,* 2023). Without validation that FCM concentrations reflect HPA axis activation and cortisol secretion into the bloodstream, the biological relevance of FCM expression may be spurious (Keay *et al.,* 2006). As such, concurrent measures of blood cortisol and FCMs are needed to calibrate the relationship between plasma cortisol concentration and subsequent FCM concentrations before drawing inference based on FCMs alone. Captive animals provide an ideal scenario to test this relationship. One method for validating the use of FCMs for non-invasive research purposes is to conduct an adrenocorticotropic hormone (ACTH) challenge. The injection of ACTH triggers the release of GCs in blood, which should then be mirrored in FCMs excreted in faeces after a species-specific time lag. Previously, studies have explored the relationship between brown bear blood cortisol and FCMs (Hunt and Wasser, 2003; White *et al.,* 2015; Dalerum *et al.,* 2020; Cattet *et al.,* 2021). Here, we expand on those previous works with larger sample sizes of bears to increase the confidence of our findings.

In this study, we assessed serum cortisol concentrations and FCM concentrations in nine captive brown bears. Our primary objectives were to (1) determine the cortisol response in serum and FCM samples following an ACTH challenge and (2) quantify the lag time between HPA activation and the expression of FCMs in brown bears.

## Materials and Methods

### Subject and materials

We conducted this study during June 2021 using nine captive brown bears (five females, four males) ranging in age from 6 to 20 years. Bears were housed at the Washington State University Bear Research, Education, and Conservation Center. For the duration of the experiment, bears were housed individually with indoor (3 × 3 × 2.5 m) and outdoor (3 × 5 × 5 m) access. The study bears had been trained previously to enter a holding crate and present a rear leg through the bars for blood collection. All bears were trained via positive reinforcement using dilute honey (in water), a method shown to not influence plasma cortisol levels (Joyce-Zuniga *et al.,* 2016). Bears were fed a commercial bear diet in the form of kibble from Mazuri (Wild Carnivore Bear Plus), apples and a small amount of meat (e.g. chicken, beef or wild game) at 7:20 am and 3:00 pm during the active season (mid-March through 1 November).

We challenged bears with 5 μg/kg Cortrosyn (Sandoz Pharmaceuticals and Amphastar Pharmaceuticals) injected intravenously (Cattet *et al.,* 2021). Bears received Cortrosyn injections between 8:40 and 9:29 am, and we collected 10 ml of blood from the metatarsal or lateral saphenous vein beginning at ~8:00 am (0 h) and then at 3, 6, 24, 48 and 72 h following injection to measure changes in serum cortisol concentrations. Once collected, the blood was centrifuged, and the serum stored at −80°C until analysed. Faecal samples were collected between 7:00 am and 8:00 pm from 24 h pre-ACTH challenge through 72 h post-ACTH challenge and placed in a −20°C freezer until shipped overnight on dry ice to Northern Michigan University where samples were stored in a −80°C freezer until analysed. Bears were under 24-h video monitoring, individuals could be identified and thus the time and source of each faecal deposition could be identified. Faecal samples deposited between 7:00 am and 8:00 pm were collected as soon as able, usually within 6 h, whilst samples deposited overnight were collected the following morning (<12 h) to avoid potential bias associated with exposure time (Stetz *et al.,* 2013; Dalerum *et al.,* 2020). Baseline serum cortisol levels were calculated as the average cortisol concentrations of plasma drawn at 0 h for each bear. FCM baselines were calculated as the average FCM concentration of samples deposited prior to the ACTH challenge (time 0). Peak blood cortisol and peak FCM concentrations were identified as the sample with the largest concentration of cortisol or cortisol metabolites following ACTH challenge.

### Faecal hormone extraction

Faecal samples were thawed at room temperature for 30 min prior to FCM extraction. We weighed 0.5 ± 0.01 g of wet faeces and placed the faeces in a 15-ml centrifuge tube with 5 ml of 80% methanol (Palme *et al.,* 2013). Samples were vortexed for 1 min and then centrifuged at 2500 g for 15 min. After being centrifuged, the supernatant was analysed immediately via ELISA kit. Final concentration was then converted from nanogrammes per millilitre into nanogrammes per gramme.

### Cortisol and cortisol metabolite assay

Serum cortisol concentrations and FCM concentrations were determined in duplicate using commercially available cortisol ELISA kits (Cortisol ELISA K003, ArborAssay, Ann Arbor, MI 48108, USA). This kit binds to immunoreactive cortisol metabolites indiscriminately, and the upper and lower detection limits of the assay were 45.4 and 27.6 pg/ml, respectively. Serum cortisol samples were brought to room temperature prior to assay, following the manufacturers protocol. For FCM samples, we modified the manufacturer’s protocol by extending the time samples were on the plate shaker to an hour and a half to increase the time for FCMs to bind to the ELISA antibodies.

### Assay validation

Faecal extracts were tested for parallelism by diluting high-FCM concentration samples (one for each sex) from 1:20 to 1:2.5 with assay buffer (Hein *et al.,* 2020). Dilutions were parallel to the standard curve (test of equal slopes, *P* > 0.30), indicating no additional substances in the extract were cross-reacting with the antibody.

All statistical analyses were conducted in R (version 4.2.2, R Core Team, 2022) using the packages ggplot2, dplyr, magrittr, tidyr and tidyverse (Wickham, 2016; Wickham *et al*., 2019, Bache and Wickham, 2022; Wickham *et al*., 2023a; Wickham *et al*., 2023b). Alpha was set at 0.05 and all tests were two-tailed. Samples were included in final analysis if intra-assay %CV was <10 and inter-assay %CV was <15. For both FCM and serum cortisol concentrations, we evaluated the change from baseline through 4 days following ACTH challenge with a repeated-measure analysis of variance (ANOVA). Additionally, we performed a two-way repeated-measures ANOVA to determine the influence of sex (male, female) and age (young, old) and the day of faeces collection/plasma collection before and after injection, in addition to the interaction between our variables (Sex:Age, Sex:Time, Age:Time). We considered young individuals as bears ≤6 years old, and old bears to be >6 years old. Next, we performed a *post hoc* Tukey’s test to determine which days of faecal collection were significantly different from one another.

To determine if FCM concentrations were correlated to blood cortisol concentrations, we conducted a linear mixed model (LMM). We used individual bear as the grouping variable in our intercept-only model random effects structure. We utilized the R packages lme4, lmerTest and sjstats (Ludecke, 2024). To conduct our LMM, we calculated the average lag time between peak blood cortisol and peak FCM concentrations. Blood cortisol concentrations were then paired with the corresponding faecal samples deposited within an hour of the adjusted time for each bear at each blood cortisol sampling time. If there wasn’t a faecal sample that fell within an hour of the adjusted time, we used the sample with the closest deposit time to the adjusted time.

**Figure 1:**
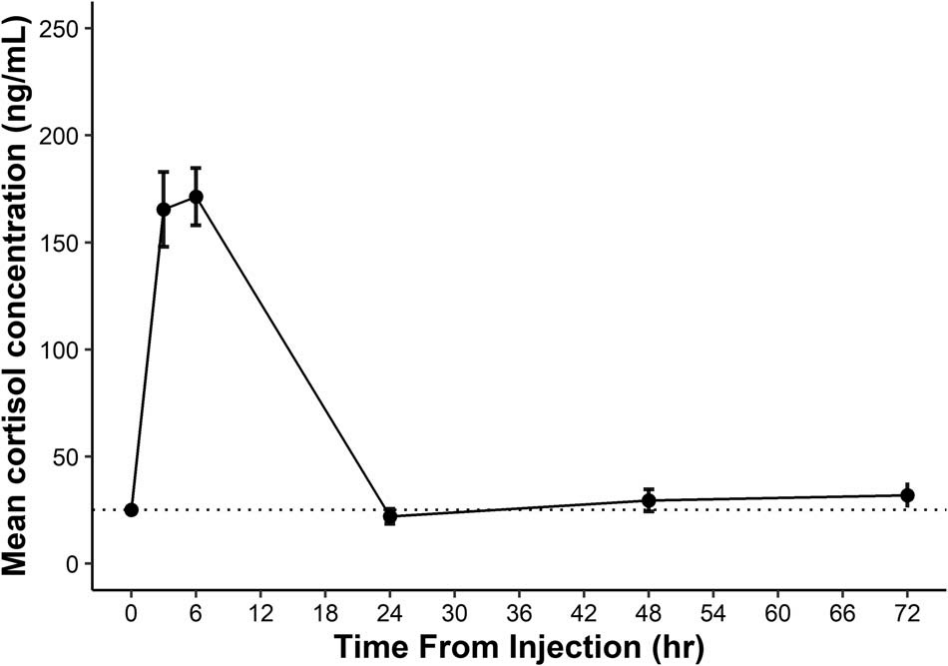
Time course of serum cortisol concentration (mean ± SEM) for nine brown bears (*U. arctos*) following injection of 5 μg/kg (i.v.) of Cortrosyn. The dotted line represents the population-level baseline concentration (24.96 ng/ml).

**Table 1:** Individual serum cortisol responses to intravenous injection of 5 μg/kg of Cortosyn in nine unanaesthetized brown bears (*U. arctos*)

			Plasma concentration		
Identification	Age (years)	Sex	Time 0 (ng/ml)	Peak (ng/ml)	Hours to peak response
Adak	6	M	28.9	160.10	3.00
Dodge	6	M	20.0	174.50	6.00
Frank	20	M	16.1	246.10	3.00
John^a^	20	M	25.4	259.80	6.00
Kio	18	F	30.5	221.20	3.00
Luna	18	F	18.8	160.70	6.00
Peeka	18	F	23.6	187.20	6.00
Willow	6	F	35.4	112.10	6.00
Zuri	6	F	26.0	152.00	6.00

a In addition to bear kibble diet, John also received Hills prescriptive digestive care diet for dogs.

## Ethical Statement

All procedures were approved by Washington State University Institutional Animal Care and Use Committee (IACUC) and we confirm that all procedures were performed in accordance with the approved IACUC ASAF #6874.

## Results

### Serum cortisol results

Following injection of Cortrosyn, serum cortisol concentrations peaked between 3 and 6 h. (Fig. 1). Serum cortisol concentrations increased from 4.5- to 10.4-fold in response to ACTH challenge above baseline levels (Table 1). Serum cortisol concentrations at 3 and 6 h post injection differed significantly from baseline cortisol (*P* < 0.001 each); however, the 3- and 6-h time period did not differ significantly from one another (*P* = 0.99). Serum cortisol concentrations returned to baseline levels by 24 h post-injection and did not differ from baseline at 72 h following injection for the remainder of the study period (Tukey’s HSD, *P* > 0.05).

**Figure 2:**
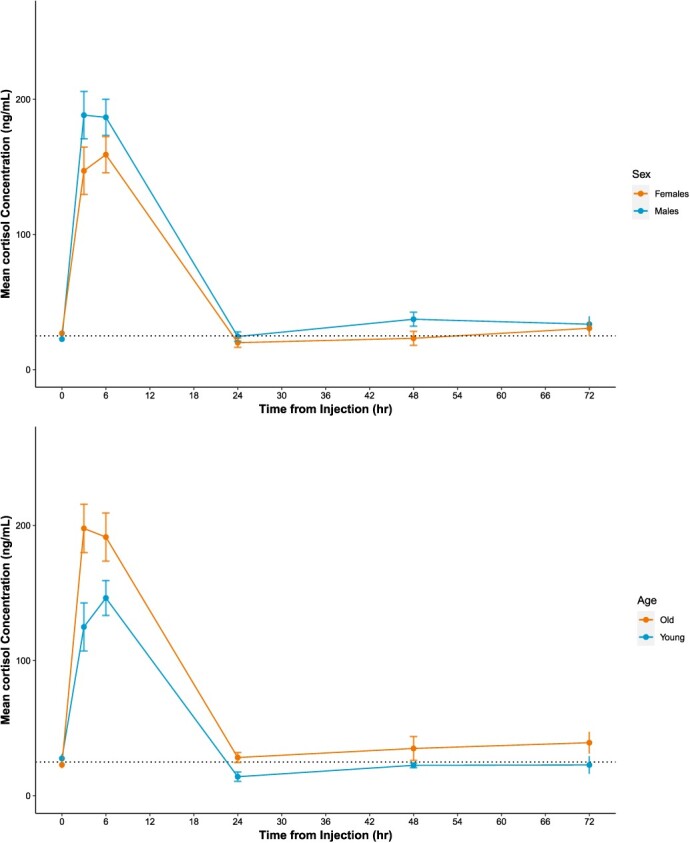
(a) Mean serum cortisol concentration (±SEM) for nine brown bears (*U. arctos*) by sex (4 males, 5 females) (b) Mean serum cortisol concentration (±SEM) of nine brown bears by age group (4 young, 5 old). Dotted line represents population-level baseline concentration (24.96 ng/ml). Bears were injected intravenously with 5 μg/kg of Cortrosyn.

Serum cortisol concentrations did not differ significantly between males and females (Two-way ANOVA: factor time, F = 58.68, *P* < 0.001; factor sex, F = 3.37, *P* = 0.07; interaction Time:Sex, F = 0.80, *P* = 0.55; Fig. 2). However, serum cortisol was significantly greater at 3 h post-injection in old versus young bears (Two-way ANOVA: factor time, F = 89.17, *P* < 0.01, factor age, F = 16.82, *P* < 0.01, interaction Time:Age, F = 3.23, *P* = 0.01). Serum cortisol concentrations did not differ between young and old bears at any other times (Tukey’s HSD, *P* > 0.05).

**Table 2:** Individual characteristics of nine brown bears (*U. arctos*) and the FCM response to intravenous injection of 5 μg/kg of Cortrosyn

			FCM concentration		
Identification	Age (years)	Sex	Time 0 (ng/g)^2^	Peak (ng/g)	Hours to peak response
Adak	6	M	3.54	172.41	13.42
Dodge	6	M	44.64	228.80	20.95
Frank	20	M	16.01	128.03	20.68
John	20	M	2.06	111.96	21.47
Kio	18	F	0.33	183.08	27.67
Luna	18	F	3.75	312.39	10.78
Peeka	18	F	1.53	146.11	27.08
Willow	6	F	9.85	115.13	22
Zuri	6	F	50.65	126.32	20.18^a^

a Zuri hours to peak response excluded the two peaks in FCM that occurred prior to injection. ^2^Time 0 value are based on mean faecal cortisol concentration of individuals prior to injection.

**Figure 3:**
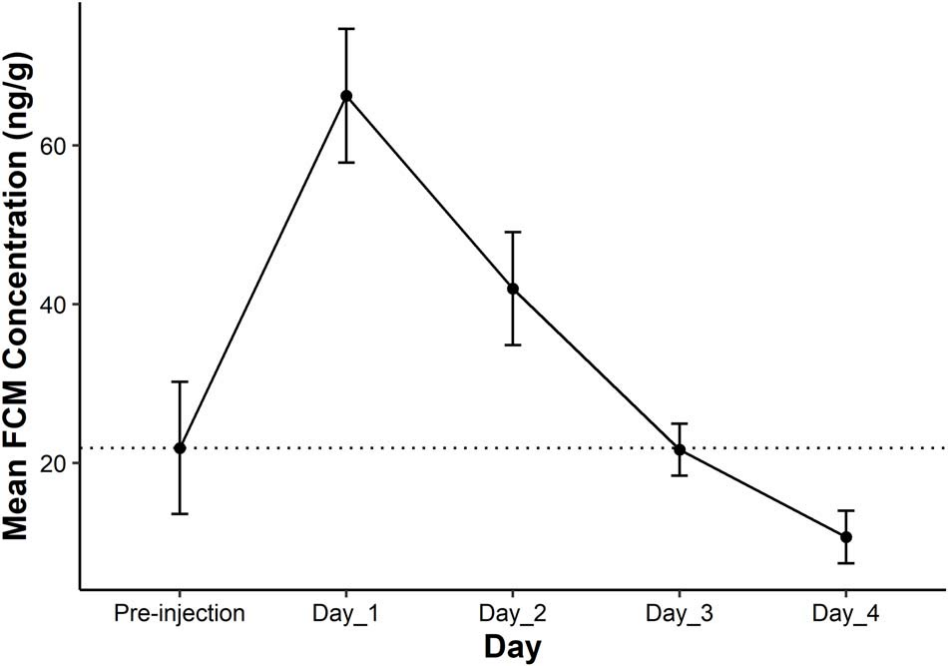
Daily mean faecal cortisol metabolite concentration (±SEM) for nine brown bears (*U. arctos*)*.* The dotted line represents population-level baseline concentration (21.90 ng/g). Bears were injected intravenously with 5 μg/kg of Cortrosyn.

### Faecal cortisol metabolite results

FCM concentration increased between 5- and 14-fold in response to ACTH challenge from baseline (Table 2). Baseline FCM concentration for all bears averaged 21.9 pg/g. On average, peak FCM occurred at 20.47 h following ACTH injection (Fig. 3). As expected, FCM patterns followed trends exhibited in serum. One individual (i.e. Zuri) had an unexpected increase in FCM during the 24 h prior to injection and on the final day of the study. Nevertheless, all animals were included in statistical analysis.

**Figure 4:**
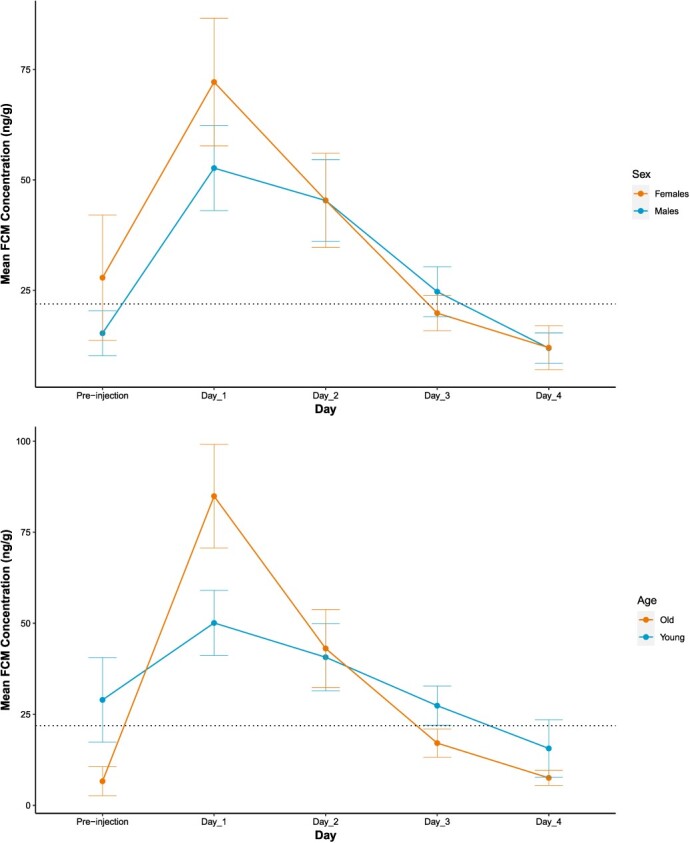
(a) Daily mean FCM concentration (±SEM) by sex (4 male, 5 female) for nine brown bears (*U. arctos*). (b) Daily mean FCM concentration (±SEM) for nine brown bears by age (4 young, 5 old). The dotted line represents population-level baseline concentration 21.90 ng/g. Bears were injected intravenously with 5 μg/kg of Cortrosyn.

FCM concentrations differed significantly from baseline during Day 1 and returned to baseline levels on Day 2 and remained at baseline levels for Days 3 and 4 (*P* < 0.01, *P* = 0.46, *P* = 0.99, *P* = 0.91, Fig. 3). However, daily mean FCM did not differ between males and females (Two-way ANOVA: factor day, F = 10.53, *P* < 0.001; factor sex, F = 0.36, *P* = 0.85; interaction Day:Sex, F = 0.23, *P* = 0.92; Fig. 4). Daily mean FCM concentrations also did not differ significantly between age groups (Two-way ANOVA: factor day, F = 10.93, *P* < 0.001; factor age, F = 0.14, *P* = 0.71) although a significant time-by-age interaction was observed (interaction Day:Age, F = 2.45, *P* = 0.04). FCM concentration differed significantly from baseline for old bears at Day 1, and returned to baseline levels for the remainder of the study; however, FCM concentrations for young bears did not differ significantly from baseline.

**Figure 5:**
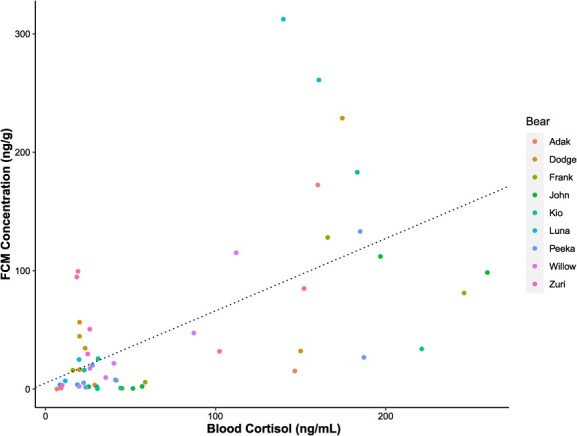
Correlation between blood cortisol concentrations (ng/ml) and FCM concentration of nine brown bears (*U. arctos*). Correlation determined by random intercept-only linear mixed model with individual bear as the grouping variable. Conditional R^2^ = 0.446, Marginal R^2^ = 0.380.

There was a positive and significant correlation between blood cortisol and FCM concentrations (Fig. 5; *P* < 0.05). Individual bears accounted for an additional 6.6% of variation in our data (Conditional R^2^ = 0.446, Marginal R^2^ = 0.380).

## Discussion

We demonstrated that brown bear FCM concentration provides an alternative and integrative measure of circulating blood cortisol concentrations to draw inferences of physiological health of an organism. Additionally, we demonstrated that blood cortisol concentrations were correlated to FCM concentration. Blood cortisol concentration accounted for 38–44% of variation in FCM concentration; however, the limitation of opportunistic faecal collection may impact that. Whilst some faecal samples were deposited within an hour of the corresponding lag time from blood samples, other individuals had multiple-hour gaps of no faecal samples. This may potentially lead to less accurate FCMs to compare with corresponding blood samples; as such, correlation between blood cortisol concentrations and FCM concentrations is likely to be a bit higher. In addition to the correlation between blood cortisol and FCMs, we found that peak FCM concentrations peaked on average 20.4 h after administering Cortrosyn. These peak times in the current study were considerably longer than the times described in White *et al.* (2015), who injected three brown bears with corticotrophin instead of Cortrosyn (see Table 3 for details). Furthermore, White *et al.* (2015) chemically immobilized their animals and conducted their study in November and December when bears differ physiologically from summer-active bears (Laske *et al.,* 2011; Ware *et al.,* 2013). Hunt and Wasser (2003) conducted an ACTH challenge with a single male and female brown bear and observed peak FCM concentrations at 22 and 32 h, respectively. The aforementioned studies may further reaffirm the need for validating lag time between secretion of blood cortisol and expression of cortisol in faeces.

**Table 3:** Summary of previous ACTH challenge studies on FCMs in a variety of bear species

Author	Species	Sample size	Drug	Mean hours to peak FCM response
White *et al.* (2015)	Brown bears (*U. arctos*)/Polar bears (*Ursus maritimus*)	3 (brown bear)/3 (polar bear)	Corticotrophin	5.63 (brown bear)/12.63 (polar bear)
Hunt and Wasser (2003)	Brown bears	2	ACTH	27
Dalerum *et al.* (2020)	Brown bears/Syrian brown bears (*Ursus arctos syriacus*)	2 (brown bear) 2 (Syrian brown bear)	ACTH	~36 (brown bear) ~48 (Syrian brown bear)

Sex did not influence blood cortisol or FCM concentrations in our study; however, sex differences in FCMs have been found in Steller sea lions (*Eumetopias jubatus*) and coyotes (*Canis lantrans*) (Mashburn and Atkinson, 2004; Stevenson *et al.,* 2018). von der Ohe *et al.* (2004) found that sex did not influence FCM concentrations of free-ranging brown bears. Thus, understanding how factors such as sex and age may influence FCM concentrations is critical for interpreting FCM concentrations as a tool for monitoring wildlife health. Although we observed an interaction between sex and time in the present study, it is likely due to small sample size.

Although we did not assess the impact of diet in the present study, diet has also been shown to influence the lag time from injection to peak FCM expression. For example, Pritchard and Robbins (1990) found that mean gut retention time for vegetation in brown bears and black bears was 7 h and whilst that for meat was 13 h, suggesting a relationship between diet composition and digestive efficiency. Zhou *et al.* (2020) found that the macronutrient composition of foods eaten by giant pandas influenced FCM concentrations. Additionally, von der Ohe *et al.* (2004) found that diet and season interacted to affect FCM concentration in free-ranging brown bears, but similar to our study.

Daily circadian rhythms that can shift seasonally may also influence cortisol concentrations in serum. Cortisol is indirectly influenced by light, leading to increases in serum cortisol concentrations during night and decreased levels during the day (Leproult *et al.,* 2001). Additionally, in brown bears, the daily means of serum cortisol have been found to vary significantly across seasons, dependent on the length of daylight (Ware *et al.,* 2013). This may be of particular importance when conducting non-invasive studies on brown bears at high and low latitudes where photoperiod may differ dramatically by season. Ware *et al.* (2013) also found that serum cortisol concentrations were higher during March compared to August. In contrast, Cattet *et al.* (2021) reported that serum and hair cortisol concentrations were higher in August compared to April. Additionally, genetics may play a role in rate of metabolism of cortisol in the blood. For example, the six related bears in our study (Adak, Dodge, Frank, John, Willow, Zuri) had lag times between ~10 and 18 h, whereas the unrelated individuals (Luna, Kio, Peeka) exhibited lag times between ~4 and 25 h. As such, relatedness may be a factor for the rate of metabolization of blood cortisol.

In summary, our work adds to the knowledge of the ecologically meaningful linkage between circulating serum cortisol and FCMs. To our knowledge, this study has utilized the largest sample size of bears, further contributing to the confidence of our findings in regard to sex and age differences in the blood cortisol and FCMs of related and unrelated individuals. Importantly, our results demonstrate that FCMs provide a potential index of stress in brown bears. The variability between lag time and magnitude of response by individuals within a controlled environment reinforces the importance of individual differences contributing to variation in the physiological response of animals after a disturbance event. Our findings contribute empirical evidence to support the application of using FCMs to non-invasively monitor long-term stress of free-ranging brown bear populations. Future studies should explore further the effect of seasonal variation in plasma and FCM concentrations, particularly as bears experience hyperphagia and emerge from torpor.

## Limitations and Considerations

Due to ethical considerations and the difficulty in defining stress, we were not able to experimentally compare the Cortrosyn-induced elevations in serum cortisol and FCM to those of an environmental stressor. This is an important consideration and one that would also be relevant to field studies where human observations and timed faecal collections would be needed to draw firm conclusions about the potential relationship between FCMs and human presence. Additionally, we used a broader cortisol detection ELISA rather than targeting a specific FCM. The cortisol ELISA kit binds to immunoreactive cortisol metabolites indiscriminately, which does not allow us to track changes in specific FCMs over the course of our study, but potentially eliminates differences in our data that could have arisen due to differences between sampling kits designed specifically for blood or faeces. Another limitation is the relatively infrequent collection times used in the present study to define the serum cortisol peak. Future studies could use more frequent blood sampling to define serum peak cortisol with greater accuracy but must also consider the implications of more frequent sampling to the well-being of the individual animals being sampled.

## Data Availability

The data gathered for this research is available in the Dryad Digital Repository at DOI: 10.5061/dryad.n8pk0p34f.
